# Native valve infective endocarditis: a rare complication of rat bite fever caused by *Streptobacillus moniliformis*

**DOI:** 10.5694/mja2.51775

**Published:** 2022-11-15

**Authors:** Caitlin Paul, Joseph O'Brien, Sarah Huffam, Daryl Ridley

**Affiliations:** ^1^ Barwon Health Geelong VIC; ^2^ Monash University Melbourne VIC; ^3^ Deakin University Geelong VIC

**Keywords:** Cardiovascular infections, Cardiac surgery, Occupational diseases, Zoonoses, Heart valve diseases

## Clinical record

A 44‐year‐old man presented to the emergency department with left knee monoarthritis; he had a history of tobacco dependence and no known cardiac disease. At presentation, he was systemically well, with normal observations and no fever. Examination revealed a moderate sized left knee effusion. Aspirate revealed 3000 × 10^6^/L leucocytes (reference interval, < 200 × 10^6^/L), negative Gram stain, and no signs of crystal arthropathy. The patient was subsequently discharged with a plan for further outpatient workup; however, he re‐presented a week later with ongoing left knee arthralgia, rigors, and a fever of 38.2°C. Diagnosed provisionally with septic arthritis, he was managed under the orthopaedic team with surgical washout and empirical intravenous (IV) ceftriaxone. Surgical joint aspirate was again culture‐negative; however, a subsequent enrichment culture and a single blood culture grew *Streptobacillus moniliformis* at 63 hours. Antibiotics were promptly switched to IV benzylpenicillin, 2.4 g 4‐hourly. Sensitivity testing was unable to be completed, and subsequent blood and synovial cultures were negative. Comprehensive history taking revealed an occupational exposure to rodent bite two weeks before index presentation through work as an animal technician.

The patient's cardiovascular examination was unremarkable, without stigmata of infective endocarditis or murmur, and a normal electrocardiogram. However, owing to concern regarding infective endocarditis, he proceeded to a transthoracic echocardiogram, which revealed a large, mobile mass fixed to the mitral valve. This was further characterised by transoesophageal echocardiography as a 13 mm × 12 mm vegetation on the anterolateral commissure, associated with progressive mitral regurgitation (Box 1). Left ventricular function was preserved with no other valvular abnormalities. Magnetic resonance imaging suggested a cerebral embolic phenomenon; however, there were no clinical sequelae of stroke. Coronary angiography, as part of surgical workup, revealed no significant coronary artery disease. Despite one week of antimicrobials, following multidisciplinary team discussion, the patient was managed surgically with a vegetectomy (Box 2) and mitral valve repair (bovine patch), curtailing the need for metallic valve replacement, followed by six weeks of IV benzylpenicillin. Despite a stable post‐operative period, the patient presented again with dyspnoea and peripheral oedema. He was found to have recurrence of moderate mitral regurgitation. In consultation with the multidisciplinary team, he was managed medically with diuretics and prolonged infection surveillance. He remains stable with improved inflammatory markers and mild mitral regurgitation on the most recent transthoracic echocardiogram.

Box 1 **Transoesophageal echocardiogram showing 13 mm × 12 mm vegetation on the posterior leaflet mitral valve**

**Figure:**
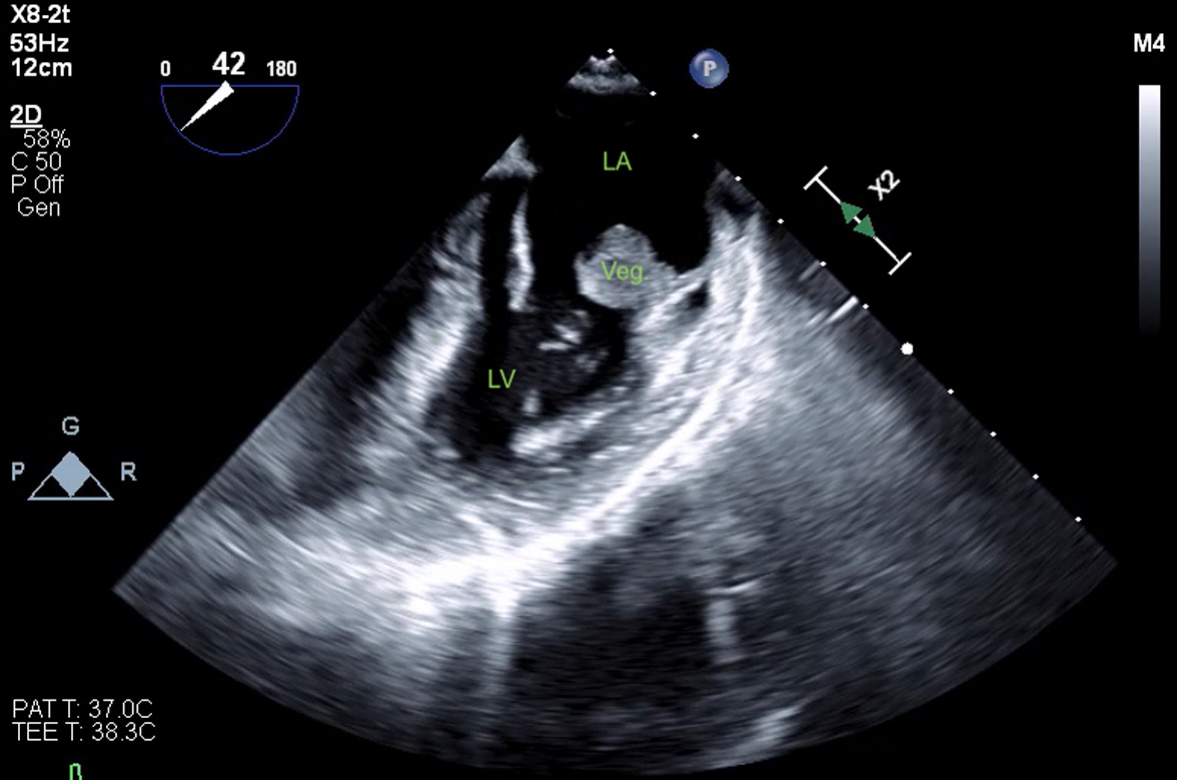
LA = left atrium; LV = left ventricle; Veg = vegetation.

Box 2 **Intraoperative image of exposed left atrium with visible vegetation (arrow) on the mitral valve**

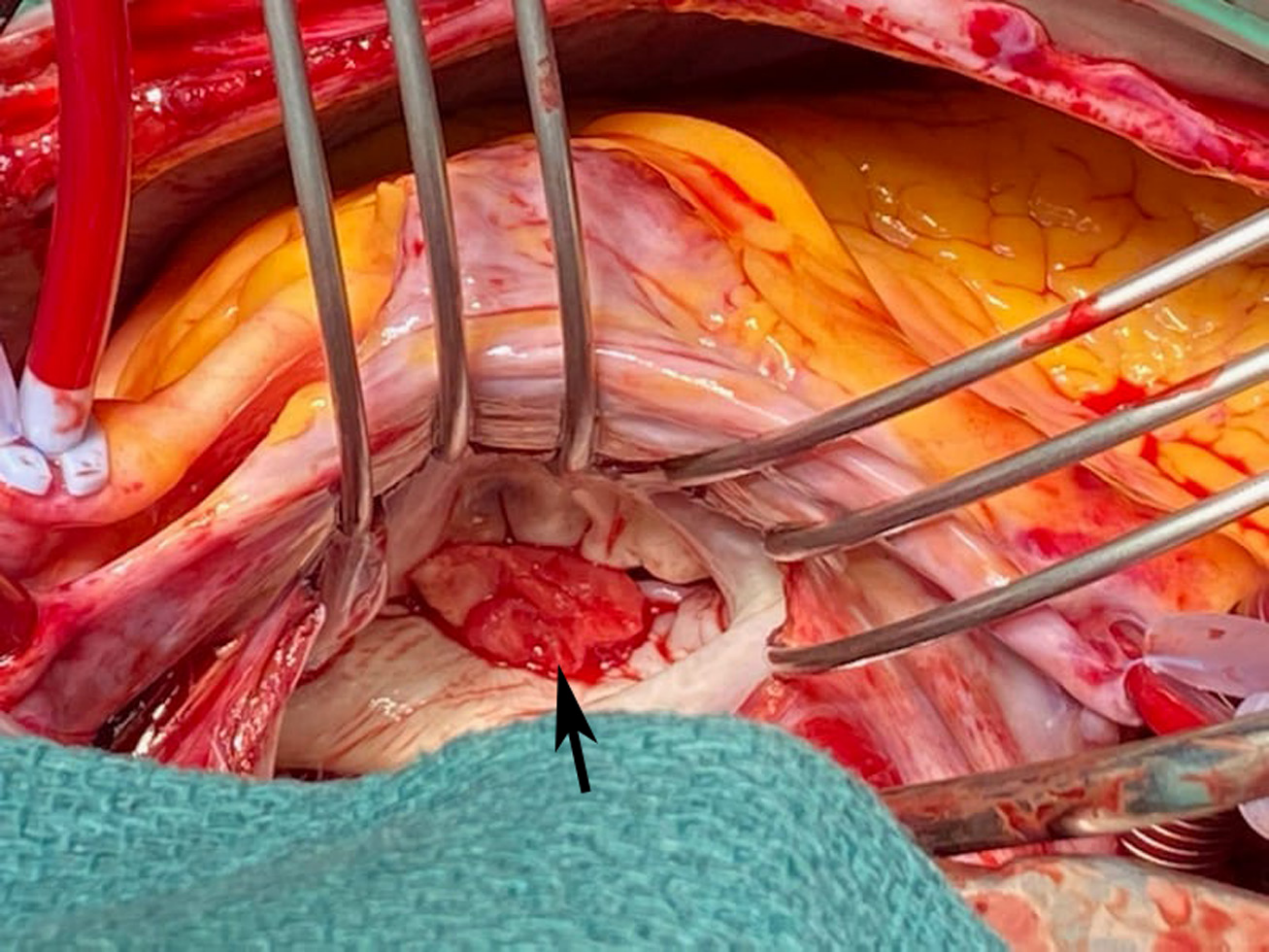

## Discussion

*Streptobacillus moniliformis* infection is rarely described in the Australian population, consistent with other developed nations.^1^ Rarer still is *S. moniliformis* infection resulting in infective endocarditis.

With near universal carriage of the bacterium in rat oropharynx, inoculation and subsequent clinical disease is estimated to occur in about 10% of people following rodent bite;^2^ the condition is therefore commonly known as rat bite fever. The history of exposure to rat bite is not always initially obtained. Contemporary risk factors include laboratory technicians, pet store workers and pet rat owners. Close contact is sufficient for disease transmission in some cases.^3^, ^4^

Symptoms usually manifest about one week after inoculation and include fever, headache, migratory polyarthritis, and maculopapular rash. Arthritis involving the knees is common^2^, ^5^ and was the initial presentation in our patient. While our patient's symptoms were consistent, the lead time to disease appeared longer than usual at nearly two weeks.^5^

Diagnosis through culture faces a number of challenges. *S. moniliformis* is a slow growing and fastidious Gram‐negative bacillus. Additionally, its growth is inhibited by polyanethol sulfonate, which is present in many commercial blood culture bottles.^2^, ^6^ Fortunately, in our patient, positive blood cultures were obtained, which greatly assisted in timely diagnosis, and appropriate antimicrobial and subsequent surgical management. In cases where culture is non‐diagnostic, a 16S ribosomal DNA polymerase chain reaction test can be performed on tissue, although it is not specific.^6^, ^7^ Empirical diagnosis and treatment is frequently necessary based on a strongly suggestive patient history.

The development of infective endocarditis is considered a rare but serious complication associated with a high mortality rate of over 25% if incompletely treated.^2^, ^6^ In most case reports there have been pre‐existing valvular abnormalities, but anatomically normal, native valve endocarditis is known to occur.^6^, ^7^ Progressive cardiac disease, including peri‐prosthetic valvular abscess and atrioventricular block, have been documented for rat bite fever in the absence of appropriate therapy.^2^, ^6^

Penicillin‐based therapy and surgical resection of diseased endocardium is the favoured approach across limited case reports, congruent with standard infective endocarditis management. In our patient, surgical management was preferred due to the size of the mitral valve vegetation and the presence of embolic sequelae. Some sources suggest four weeks of penicillin therapy plus two weeks of gentamicin therapy as the preferred antimicrobial strategy,^2^ whereas others recommend six continuous weeks of antimicrobials.^6^ Because of the highly virulent nature of *S. moniliformis*, we would suggest six weeks of antimicrobials; benzylpenicillin monotherapy was favoured for our patient owing to the risk of gentamicin ototoxicity and nephrotoxicity.

## Lessons from practice

- Identifying exposure to fastidious organisms is imperative to aid a diagnosis of infective endocarditis.
- It is important to investigate for infective endocarditis using transthoracic echocardiography despite the absence of clinical signs when a high risk organism such as *Streptobacillus moniliformis* has been identified.
- The apparent aggressive nature of *S. moniliformis* should lead to strong consideration of the use of prolonged intravenous antimicrobials.

## Open access

Open access publishing facilitated by Deakin University, as part of the Wiley – Deakin University agreement via the Council of Australian University Librarians.

## Competing interests

No relevant disclosures.

## Provenance

Not commissioned; externally peer reviewed.

## References

[1] Adam JK , Varan AK , Pong AL , McDonald EC . Notes from the field: fatal rat‐bite fever in a child – San Diego County, California, 2013. MMWR Morb Mortal Wkly Rep 2014; 63: 1210‐1211. 25522092 PMC5779525

[2] Elliot SP . Rate bite fever and *Streptobacillus moniliformis* . Clin Microbiol Rev 2007; 20; 13‐22. 17223620 10.1128/CMR.00016-06 PMC1797630

[3] Torres L , López AI , Escobar S , et al. Bacteremia by *Streptobacillus moniliformis*: first case described in Spain. Eur J Clin Microbiol Infect Dis 2003; 22: 258. 12709841 10.1007/s10096-003-0891-9

[4] Swan CD , Koirala A , Samarasekara H . *Streptobacillus moniliformis* bacteraemia and septic arthritis in a child. J Paediatr Child Health 2022; 58: 1465‐1467. 34927295 10.1111/jpc.15855

[5] Dendle C , Woolley IJ , Korman TM . Rat‐bite fever septic arthritis: illustrative case and literature review. Eur J Clin Microbiol Infect Dis 2006; 25: 791. 17096137 10.1007/s10096-006-0224-x

[6] Winther M , Jense HS , Tarpgaard I , Nielsen H . Case report: a fatal case of aortic and mitral valve endocarditis caused by *Streptobacillus monoliformis* . Eur Heart J Case Rep 2020; 4: 1‐6. 10.1093/ehjcr/ytaa254 PMC7780487 33426458

[7] Crofton, KR , Ye J , Lesho EP . Severe recurrent *Streptobacillus moniliformis* endocarditis in a pregnant woman, and review of the literature. Antimicrob Resist Infect Control 2020; 9: 119. 32727581 10.1186/s13756-020-00789-4 PMC7391614

